# Changes in Clinical Parameters During Low-Frequency Outpatient Pulmonary Rehabilitation for Male Patients With Chronic Obstructive Pulmonary Disease

**DOI:** 10.7759/cureus.81413

**Published:** 2025-03-29

**Authors:** Shota Kotani, Junpei Oba, Kunihiko Anami, Takeshi Yamazaki, Jun Horie

**Affiliations:** 1 Department of Physical Therapy, Faculty of Rehabilitation Science, Kobe International University, Kobe, JPN; 2 Graduate School of Health Science, Kyoto Tachibana University, Kyoto, JPN; 3 Department of Rehabilitation, Osaka Anti-Tuberculosis Association Osaka Fukujuji Hospital, Osaka, JPN; 4 Department of Rehabilitation, Faculty of Health Sciences, Naragakuen University, Nara, JPN; 5 Department of Physical Therapy, Faculty of Health Sciences, Kyoto Tachibana University, Kyoto, JPN

**Keywords:** cognitive function, copd patients, low frequency, mild cognitive impairment (mci), pulmonary rehabilitation, rehabilitation effects

## Abstract

Introduction: Chronic obstructive pulmonary disease (COPD) is a progressive condition characterized by systemic inflammation, which leads to impaired respiratory function and a wide range of comorbidities. Mild cognitive impairment (MCI) has been identified as a precursor to dementia and is more prevalent in patients with COPD than in the general population. Pulmonary rehabilitation (PR) is recognized as the standard therapy for COPD in international guidelines; however, the frequency and long-term effects of PR remain insufficiently explored. We aimed to evaluate changes in MCI, physical function, physical activity, activities of daily living (ADL), mental health, and health-related quality of life (HRQOL) in male COPD patients with low-frequency outpatient PR once a month over a two-year period.

Methods: This retrospective, longitudinal study was conducted at a respiratory disease specialty hospital between April 2018 and September 2024. A total of 80 male patients with COPD were enrolled, of whom eight were excluded based on the exclusion criteria. Additionally, 51 patients who could not maintain PR for two years were also excluded, leaving 21 participants for the final analysis. Assessments included baseline characteristics, body composition, physical function, physical activity, cognitive function, frontal lobe function, HRQOL, ADL, and mental health. Outpatient PR sessions, conducted monthly in conjunction with physician consultations, included a 40-minute program consisting of exercise therapy, ADL guidance, and patient education.

Results: Significant reductions were observed in step counts (p = 0.048, d = 0.46) between baseline and two years. Significant reductions were observed in the ADL indices, specifically in the NRADL subdomains of movement speed (p = 0.007, d = -0.59), breath of shortness (p = 0.003, d = -0.64), oxygen flow (p = 0.035, d = -0.46), and the total score (p = 0.006, d = -0.46). No significant changes were observed in cognitive function, frontal lobe function, HRQOL, or psychological metrics. Reductions in the frequency of exacerbations and hospitalizations were observed in some patients, suggesting the stabilization of symptoms, particularly in specific Global Initiative for Chronic Obstructive Lung Disease (GOLD) categories and stages.

Discussion: While low-frequency PR over two years showed limited efficacy in maintaining physical activity levels and ADL, it contributed to symptom stabilization and a reduction in acute exacerbations. These findings suggest that monthly PR sessions are insufficient to achieve significant improvements in cognitive function or physical activity. High-frequency interventions may be required to optimize outcomes. Additionally, the challenges in maintaining long-term adherence to PR highlight the potential benefits of integrating home-based or telerehabilitation approaches into comprehensive intervention strategies.

## Introduction

Chronic obstructive pulmonary disease (COPD) is a systemic inflammatory disease with various complications [1,2]. One cause is cognitive dysfunction. Mild cognitive impairment (MCI) is an intermediate condition between normal aging and dementia. Therefore, it is positioned in the pre-dementia stage. The prevalence of MCI in adults aged 65 years is 10%-20% [3]. In contrast, the MCI complication rate in patients with COPD has been reported to be 36% [4]. The prevalence of MCI in patients with COPD is higher than that in the general older population. In addition, 5%-17% of patients with MCI progress to dementia within one year but may recover normally [5,6]. Therefore, detection of MCI complications in patients with COPD at an early stage is necessary, as this is not a condition that can be taken lightly. Investigating the effect of pulmonary rehabilitation (PR) on MCI is valuable.

PR is considered a standard nondrug therapy [7]. In addition, although there are scattered studies of PR interventions, a Cochrane review of their frequency found that most PR programs were eight or 12 weeks in duration, with an overall range of four to 52 weeks [8]. Spruit et al. reported that the frequency of outpatient PR programs in hospitals is two or three days per week [9].

The advantages include high program implementation rates and PR effectiveness owing to short-term intensive PR programs. However, the disadvantage is that after the PR program is completed, if there is no follow-up to maintain the effects, the improvement reverts to before the PR program was implemented [10,11].

The PR Statement in Japan states that “pulmonary rehabilitation should be a seamless intervention” [12]. Few previous studies have shown the long-term continuation rates of PR at home or in outpatient settings [13].

Outpatient follow-up is important for a seamless intervention. However, only 50.5% and 18.0% of the patients with chronic respiratory disease in the later stages of life continued to attend the outpatient PR program after completion of the first and second years, respectively. This was significantly poorer than 79.5% in the first year and 66.2% in the second year for older adults in the previous period [14]. The reasons for this include self-interruption, exacerbation of the underlying disease, exacerbation of diseases other than pulmonary disease, and difficulty in getting to the hospital. Therefore, outpatient PR is difficult to sustain, but if continued, it is effective in maintaining exercise tolerance [13,14].

Although reports on the effects of PR on the maintenance of physical function are available, only a few reports are found on the effects of low-frequency outpatient PR on cognitive function. The frequency and timing of interventions are critical in long-term outpatient PR. Patients visit their doctors once a month. Outpatient PR in conjunction with this visit allows for long-term interventions. Thus, in this study, we aimed to determine the changes in clinical indicators resulting from a lower frequency of outpatient PR under usual care in male COPD patients.

## Materials and methods

Study design

In this retrospective longitudinal study, we analyzed the indicators of outpatient respiratory rehabilitation at baseline and at two years post-intervention.

Patient enrollment

**Figure 1:**
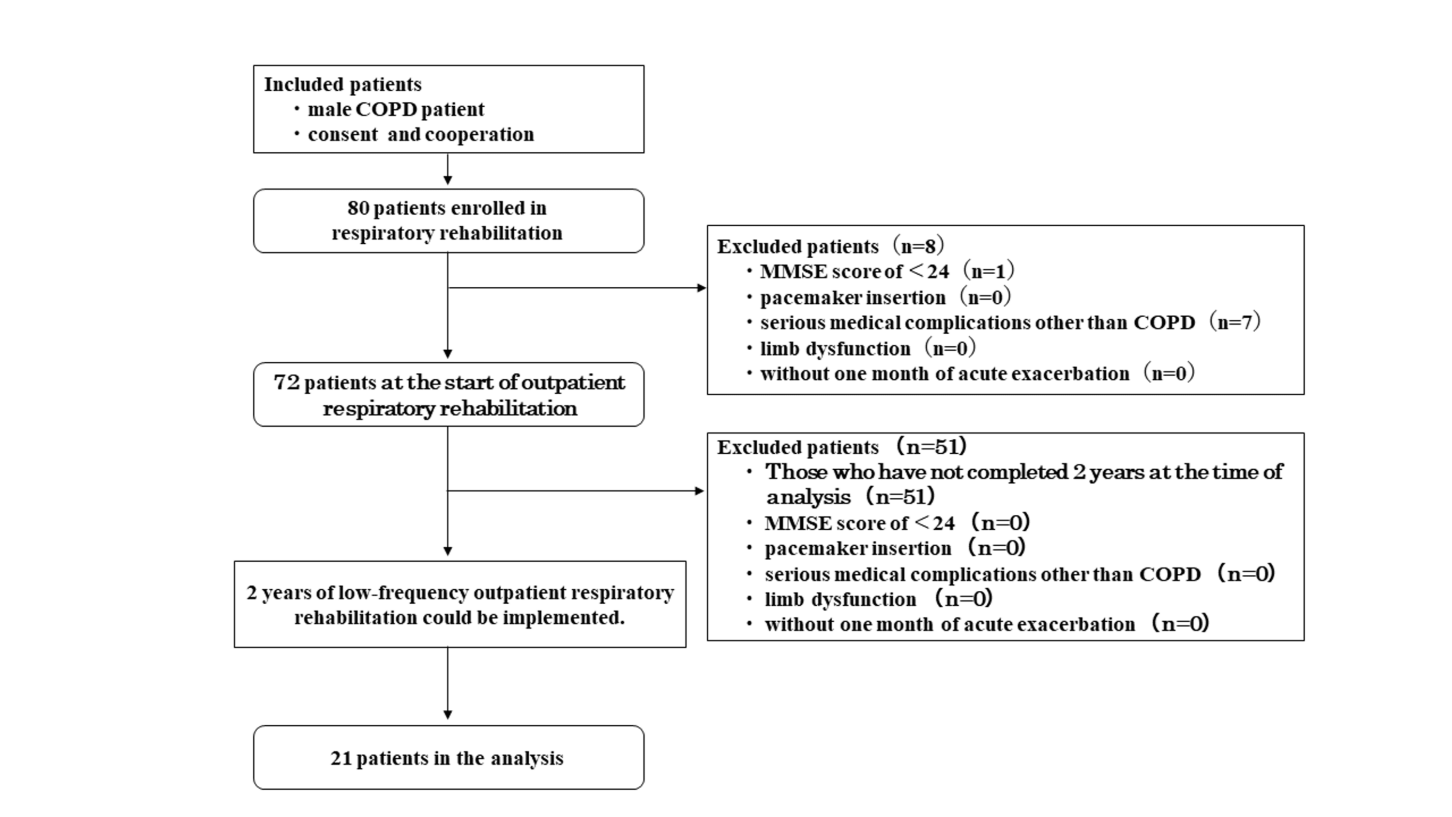
Flowchart of the analysis

Eighty male patients with COPD enrolled in outpatient respiratory rehabilitation at a hospital specializing in respiratory diseases between April 2018 and September 2024 were included in the study. The inclusion criteria were male COPD patients who were in a stable condition, at any disease stage, able to provide consent and cooperate, and could be evaluated during outpatient PR under usual care for two years. The exclusion criteria were as follows: suspected dementia with a Mini-Mental State Examination (MMSE) score of <24, pacemaker insertion, serious medical complications other than COPD, impaired mobility due to cerebrovascular disease sequelae, or limb dysfunction due to motor system disease, patients within one month of acute exacerbation, and no two-year lapse at the time of analysis (Figure 1).

Measurement indices

Measures included basic characteristics, body composition, physical function, physical activity, health-related quality of life (HRQOL), activities of daily living (ADL), mental health, cognitive function, and frontal lobe function. Cognitive function and frontal lobe function were set as the primary outcomes, while the other measures were considered secondary outcomes.

Indicators of demographic characteristics included sex, modified Medical Research Council dyspnea scale (mMRC scale) score, age, height, weight, forced expiratory volume in one second/forced vital capacity (FEV_1_/FVC), %FEV_1_, %vital capacity (%VC), Global Initiative for Chronic Obstructive Lung Disease (GOLD) stage, GOLD category, inhaled medications, comorbidities, years of education, smoking history, and the use of home oxygen therapy (HOT).

Cognitive function was assessed using the MMSE score and the Japanese version of the Montreal Cognitive Assessment (MoCA-J) score. The index of frontal lobe function was the FAB score.

Body composition indices were measured using a body composition analyzer (InBody270®; InBody, Japan). The body mass index (BMI), muscle mass, skeletal muscle mass, and skeletal muscle mass index (SMI) were measured.

Physical function indices were measured based on grip strength (Digital Grip Strength Scale®; Takei Kiki Kogyo, Japan) and isometric maximum knee extension strength (μTas F-1®, Anima, Japan) for muscle strength, incremental shuttle waking distance (ISWD) for exercise tolerance, and Mini Nutritional Assessment-Short Form (MNA) scores for nutrition. Grip strength and isometric maximum knee extension muscle strength were measured twice on each side, and the maximum value was used. The patients were placed in a sitting position with their lower legs drooped to measure knee extensor muscle strength. The Tas F-1 sensor was placed directly above the internal and external parts of the lower leg. A fixation belt was then connected to a post at the back and adjusted such that the knee joint was at 90° of flexion when extended during the measurement. The patient was taught to extend the knee with maximum force for three seconds during the measurement.

Physical activity indices were measured using a triaxial accelerometer (Active Style Pro; Omron). The number of steps, walking time, amount of walking exercise (Ex), amount of daily activity Ex, total Ex, weekly Ex, and activity time over the three metabolic equivalents (METs) were measured.

The HRQOL index was the score of the COPD assessment test (CAT), and the ADL index was the score of movement speed, shortness of breath, oxygen flow, continuous walking distance, and the Nagasaki University Respiratory ADL Questionnaire (NRADL).

Measurement of physical activity and data processing

Daily measurement data for physical activity were obtained on days with at least six hours of activity, avoiding the middle of the night when older adults are less active [15]. The measurement period was one month from the date of evaluation. Patients were instructed to wear the device on the waist of their pants from the time they got up until they went to bed. In addition, they were instructed to remove it while bathing and going to bed. Moreover, the influence screen of the triaxial accelerometer was set to display only time.

Data were processed using a software (activity meter application version 2.9®, OMRON, Japan) dedicated to the triaxial accelerometer. Additionally, data were collected only on days when the triaxial accelerometer was worn for at least 360 min/day [15]. As the measurement period was set at one month, hospital visits and exercise therapy by rehabilitation staff were regarded as physical activity and consequently included in the analysis.

Low-frequency PR in routine care

Low frequency was defined as outpatient respiratory rehabilitation performed once a month on the same day as the physician's visit, and the duration of each outpatient respiratory rehabilitation session was 40 minutes. The regimen consisted of strength training of the upper and lower limbs and trunk under body weight, aerobic exercise using a bicycle ergometer, ADL instructions, and patient education, as generally performed in respiratory rehabilitation [10].

Statistical analyses

To adjust for missing values, the mean value calculated from the data without missing values was substituted. The χ2 independence test was used to relate the measured indicators between baseline and two years after PR. After normality testing was performed using the Shapiro-Wilk test, measurements between baseline and two years after PR were compared using corresponding t-tests or the Wilcoxon signed-rank test. The statistical significance level was set at 5%, and IBM SPSS Statistics for Windows, Version 29 (Released 2023; IBM Corp., Armonk, New York, United States) was used for the statistical analysis.

Ethical considerations

A written informed consent was obtained from all participating adults before enrollment. This study was approved by the Research Ethics Committee of Kyoto Tachibana University (Approval No. 21-48).

## Results

Eighty male COPD patients were enrolled. Finally, 21 patients were included in the analysis. Baseline age was 76.4 ± 7.3 (two years, %FEV1: 61.2 ± 27.5%; two-year age: 78.4 ± 7.3 years, %FEV1: 57.8 ± 18.1%). In the GOLD staging system, the baseline values were stage I (0), stage II (16), stage III (4), and stage IV (1). After two years, stage I, 2; stage II, 14; stage III, 4; and stage IV, 1 showed a significant difference (p < 0.048, d < 0.01) (Table 1).

**Table 1:** Baseline characteristics of the respiratory rehabilitation at baseline and after two years % FEV1: %forced expiratory volume in one second; %VC: %vital capacity; FEV1/FVC: forced expiratory volume in one second/forced vital capacity; BI: Brinkman Index; GOLD: Global Initiative for Chronic Obstructive Lung Disease; HOT: home oxygen therapy; ICS: inhaled corticosteroid; LABA: long-acting β-agonist; LAMA: long-acting muscarinic antagonist; mMRC scale: Modified Medical Research Council Dyspnea Scale Note: The corresponding t-test or Wilcoxon signed-rank test was performed to determine the difference between baseline and at two years. The χ² test for independence was used to assess nominal variables. Statistical significance was determined by p-values and t-values and χ²-values, and effect sizes were reported as appropriate. Data are presented as means (SD) a: The analysis was conducted using the chi-square test for independence

（n= 21)	baseline	After two years	t-value	p-value	Effect size
mMRC scale^ a^					
Grade 0 (n,%)	3 (14.3)	3 (14.3)	84.000	0.84	<0.01
Grade 1 (n,%)	7 (33.3)	6 (28.6)			
Grade 2 (n,%)	8 (38.1)	5 (23.8)			
Grade 3 (n,%)	3 (14.3)	4 (19.0)			
Grade 4 (n,%)	0 (0)	3 (14.3)			
Age (years)	76.4 ± 7.3	78.4 ± 7.3	-41.000	<0.001	-8.95
Height (cm)	164.9 ± 6.8	164.9 ± 6.8	n.s.	n.s.	n.s.
Weight (kg)	59.0 ± 10.1	57.8 ± 10.4	1.258	0.223	0.27
FEV_1_/FVC (%)	55.6 ± 15.6	52.9 ± 10.6	0.940	0.358	0.21
%FEV_1_ (%)	61.2 ± 27.5	57.8 ± 18.1	0.843	0.409	0.18
%VC (%)	85.8 ± 20.3	87.8 ± 12.0	-0.623	0.540	-0.12
GOLD stage ^a^					
Stage Ⅰ (n,%)	0 (0)	2 (9.5)	18.000	<0.001	0.02
Stage Ⅱ (n,%)	16 (76.2)	14 (66.7)			
Stage Ⅲ (n,%)	4 (19.0)	4 (19.0)			
Stage Ⅳ (n,%)	1 (4.7)	1 (4.7)			
GOLD category^ a^					
Category A (n,%)	9 (42.9)	9 (42.9)	8.474	0.076	0.04
Category B (n,%)	5 (23.8)	9 (42.9)			
Category E (n,%)	7 (33.3)	3 (14.3)			
Inhalation medication ^a^					
LABA or LAMA (n,%)	1(4.7)	1(4.7)	91.636	<0.001	<0.01
LABA or LAMA + ICS (n,%)	3 (14.3)	4 (19.0)			
LABA + LAMA (n,%)	7 (33.3)	4 (19.0)			
LABA + LAMA + ICS (n,%)	5 (23.8)	7 (33.3)			
No inhaler (n,%)	5 (23.8)	5 (23.8)			
Complications ^a^					
Hypertension (n,%)	7 (33.3)	7 (33.3)	1.564	0.305	0.14
Hyperlipidemia(n,%)	2 (9.5)	1 (4.7)	0.124	1.000	n.s.
Heart disease (n,%)	1 (4.7)	3 (14.3)	0.198	1.000	n.s.
Diabetes mellitus (n,%)	1 (4.7)	1 (4.7)	0.059	1.000	n.s.
Cancer (n,%)	1 (4.7)	1 (4.7)	0.059	1.000	n.s.
History of smoking^ a^					
Never smoked (n,%)	1 (4.7)	1 (4.7)	21.058	<0.001	1.00
Former smokers (n,%)	19 (90.5)	18 (85.7)			
Current smokers (n,%)	1(4.7)	2(9.5)			
BI (number of cigarettes smoked*years of smoking)	1397.0 ± 667.9	1549.0 ± 622.0	-0.920	0.214	-0.29
HOT^ a^					
User (n,%)	5 (23.8)	10 (47.6)	2.898	0.141	0.19
Non-user (n,%)	16 (76.2)	11 (52.4)			
Number of acute exacerbations in the past year (times/year)	0.3 ± 0.6	0.2 ± 0.4	n.s.	0.405	-0.18
Number of hospitalizations in the past year (times/year)	0.3 ± 0.5	0.1 ± 0.3	n.s.	0.157	-0.31

Table 2 shows the results of the comparison of body composition, physical function, and physical activity at baseline and at two years after respiratory rehabilitation. Physical activity measures, number of steps (p = 0.048, d = 0.46), and activity time of less than three METs (p = 0.041, d = 0.13) were significantly lower at two years than at baseline. However, no significant differences were observed in body composition, physical function, or other measures of physical activity.

**Table 2:** Comparison between body composition, physical function, and physical activity at baseline and two years after respiratory rehabilitation BMI: Body Mass Index; Ex: Exercise; ISWD: incremental shuttle walking distance; MNA: Mini-Nutritional Assessment-Short Form; METs: metabolic equivalent; SMI: skeletal muscle Note: The corresponding t-test or Wilcoxon signed-rank test was performed to determine the difference between baseline and at two years.  Statistical significance was determined by p-values and t-values, and effect sizes were reported as appropriate. Data are presented as means (SD)

（n = 21)	Baseline	After two years	t-value	p-value	Effect size
BMI (kg/㎡)	22.1 ± 4.5	21.1 ± 3.7	1.416	0.172	0.30
Muscle mass (kg)	40.8 ± 4.9	39.9 ± 5.4	1.637	0.117	0.36
Body fat mass (kg)	15.8 ± 1.4	14.8 ± 1.4	1.021	0.320	-0.22
Skeletal muscle mass (kg)	23.8 ± 3.9	25.0 ± 3.8	1.793	0.283	-0.29
Basal metabolism (kcal)	1300.4 ± 112.0	1283.2 ± 124.1	1.417	0.172	0.31
SMI (kg/㎡)	6.6 ± 0.7	6.4 ± 0.7	1.795	0.088	0.39
Grip (kg)	31.9 ± 6.3	30.0 ± 5.9	2.035	0.055	0.44
Knee extension (kgf)	32.9 ± 9.9	33.0 ± 9.6	-0.017	0.987	<0.01
ISWD (m)	360.5 ± 157.9	354.7 ± 170.2	0.441	0.664	0.10
MNA (points)	11.8 ± 2.0	11.7 ± 1.7	0.335	0.741	0.07
Step (step/days)	2802.2 ± 2060.3	2086.0 ± 1711.5	2.104	0.048	0.46
Walking time (min/days)	46.5 ± 25.3	33.8 ± 20.7	1.977	0.021	0.55
Amount of walking Ex (Ex/days)	0.9 ± 0.9	0.6 ± 0.7	n.s.	0.122	-0.34
Amount of life activities Ex (Ex/days)	1.3 ± 0.6	1.1 ± 0.6	1.977	0.062	0.43
Amount of total Ex (Ex/days)	2.2 ± 1.3	1.7 ± 1.2	2.009	0.058	0.44
Amount of weekly Ex (Ex/weeks)	15.5 ± 9.1	12.2 ± 8.4	2.019	0.057	0.44
<3METs activity time (min/days)	630.1 ± 130.0	611.8 ± 136.3	n.s.	0.768	-0.06
≧3METs activity time (min/days)	37.7 ± 21.7	28.8 ± 20.0	2.181	0.549	0.48

Table 3 shows the results of the comparison of cognitive and frontal lobe function, HRQOL, ADL, and mental health at baseline and two years after PR. The ADL measures of NRADL movement speed (p = 0.007, d = -0.59), shortness of breath (p = 0.003, d = -0.64), oxygen flow (p = 0.035, d = -0.46), and total (p = 0.006, d = -0.46) were significantly lower at two years than at baseline. However, no significant differences were observed in the measures of cognitive and higher brain function, HRQOL, other ADLs, or mental health.

**Table 3:** Comparison of cognitive and frontal lobe function, HRQOL, ADL, and mental health at baseline and two years after respiratory rehabilitation ADL: activity of daily living; HRQOL: health-related quality of life; CAT: COPD assessment test; FAB: frontal assessment battery; HADS: Hospital Anxiety and Depression Scale; MMSE: Mini-Mental State Examination; MoCa-J: Japanese version of the Montreal Cognitive Assessment; NRADL: Nagasaki University Respiratory ADL Questionnaire Note: The corresponding t-test or Wilcoxon signed-rank test was performed to determine the difference between baseline and at two years.  Statistical significance was determined by p-values and t-values, and effect sizes were reported as appropriate. Data are presented as means (SD)

（n = 21)	Baseline	After two years	t-value	p-value	Effect size
MoCA-J (points)	23.9 ± 4.0	24.3 ± 4.1	-0.360	0.722	-0.08
MMSE (points)	28.5 ± 1.8	28.3 ± 2.3	n.s.	0.122	-0.03
FAB (points)	14.9 ± 2.0	14.8 ± 1.7	0.269	0.791	0.06
CAT (points)	15.5 ± 6.7	15.1 ± 6.2	0.340	0.738	0.08
NRADL					
Movement speed (points)	26.6 ± 6.7	24.2 ± 3.7	n.s.	0.007	-0.59
Shortness of breath (points)	27.0 ± 2.7	24.3 ± 3.9	n.s.	0.003	-0.64
Oxygen flow (points)	24.0 ± 11.1	20.8 ± 11.6	n.s.	0.035	-0.46
Continuous walking distance (points)	6.8 ± 3.4	6.7 ± 3.6	n.s.	0.929	-0.02
Total (points)	84.2 ± 16.6	76.5 ± 18.1	n.s.	0.006	-0.46
HADS					
Anxiety (points)	3.3 ± 2.4	3.3 ± 3.4	<0.001	1.000	<0.01
Depression (points)	6.4 ± 3.0	7.8 ± 4.2	n.s.	0.085	-0.38
Total (points)	9.7 ± 4.6	11.1 ± 6.5	n.s.	0.400	-0.18

## Discussion

Although respiratory rehabilitation should be a seamless intervention [12], maintaining outpatient respiratory rehabilitation is challenging [14]. Therefore, the adequate timing and frequency of interventions were determined in this study. Male COPD patients underwent once-monthly outpatient respiratory rehabilitation under usual care for two years to facilitate the examination of the changes in MCI and other clinical measures. The degree of shortness of breath remained unchanged over the two-year period. In addition, in the GOLD category, the percentage of category E patients decreased from 33.3% to 14.3% due to a decrease in the number of exacerbations and hospitalizations in the past year.

COPD is one of the leading causes of death worldwide, with exacerbation periods of rapidly worsening symptoms scattered throughout the natural course of the condition [16]. In addition, a systematic review of acute COPD exacerbations and readmission rates reported that COPD readmissions within 30, 60, 90, 180, and 365 days after exacerbation were 11%, 17%, 17%, 30%, and 37%, respectively. The reported risk factors include the male sex, number of hospitalizations in the previous year, and duration of hospitalization [17]. Further worsening of exacerbation and rehospitalization rates have been shown with the passage of time since the exacerbation. Furthermore, the risk of death increases with an increase in the number of exacerbations per year [18,19]. However, in the present study, a positive trend was observed over the two-year course of the study. It was suggested that PR, although as infrequent as once a month, may have helped bring about certain benefits.

During the two years of low-frequency outpatient respiratory rehabilitation, patients had significantly lower physical activity and ADL measures than at baseline. Regarding ADL, the progression of COPD pathology may be a contributing factor. COPD is a progressive systemic inflammatory disease, and a combination of age-related changes can cause symptoms to progress [20]. A statistically significant decrease in oxygen flow was observed in the NRADL between baseline and the two-year follow-up assessment. Increased oxygen flow or the initiation of HOT were likely factors, and disease progression was inferred. Therefore, shortness of breath, movement speed, and total score were significantly lower at two years than at baseline.

Physical activity is an important factor in COPD [21,22], but patients with COPD have been shown to be less physically active than healthy individuals [23,24]. PR increases physical activity in patients with COPD. However, other studies have reported no increase in physical activity. However, these results have not been consistent [25-27]. Thus, the study showed that improvement in the amount of physical activity in male COPD patients over a two-year period is difficult with low-frequency outpatient respiratory rehabilitation once a month. Therefore, this study suggests the need to reconsider intervention frequency and methods.

However, the patients were able to maintain body composition, physical function, cognitive function, frontal lobe function, HRQOL, and mental health measures in the clinical measures over the two-year study period. Aging is a progressive decline in homeostasis that increases the risk of disease development and death. A close relationship between aging and chronic inflammatory diseases has been demonstrated [28]. The addition of degenerative aging to the COPD-specific pathology of systemic inflammation is likely to lead to a further decline in clinical measures. Approximately 5%-17% of patients with MCI progress to dementia each year [5,6]. The fact that low-frequency outpatient respiratory rehabilitation maintained cognitive and frontal lobe function for two years is a significant achievement [5]. The results are expected to help reconsider intervention methods to achieve the “seamless intervention” described in the statement. In addition, HRQOL and mental health, which are problems in COPD patients with complications, could be maintained for two years with low-frequency outpatient respiratory rehabilitation. To achieve long-term improvements in clinical outcomes, future research should investigate optimal frequencies, intensities, and durations of intervention, making this an ongoing area of study.

One limitation of this study is the small sample size. Additionally, the two-year progression of the MCI group and the non-MCI group could not be adequately analyzed. Furthermore, since the study excluded subjects who were unable to undergo respiratory rehabilitation for two years, there is a possibility of selection bias. In the future, increasing the sample size will be essential to comprehensively analyze the progression of both groups. Addressing these limitations will be an important focus of future research.

## Conclusions

In conclusion, low-frequency outpatient respiratory rehabilitation, performed once a month for two years, demonstrated a positive effect on maintaining cognitive and frontal lobe function in male COPD patients, despite a decline in physical activity and ADL measures. While the intervention did not significantly improve physical activity or overall quality of life, it helped stabilize key clinical indicators, including cognitive function, suggesting the potential for a “seamless intervention” approach in COPD care. These findings highlight the importance of exploring optimal intervention frequencies and methods to achieve long-term improvements, with future research needed to further investigate the effects on both MCI and non-MCI patient groups.
